# The Genetic Linkage Map of the Medicinal Mushroom *Agaricus subrufescens* Reveals Highly Conserved Macrosynteny with the Congeneric Species *Agaricus bisporus*

**DOI:** 10.1534/g3.115.025718

**Published:** 2016-02-26

**Authors:** Marie Foulongne-Oriol, Manuela Rocha de Brito, Delphine Cabannes, Aurélien Clément, Cathy Spataro, Magalie Moinard, Eustáquio Souza Dias, Philippe Callac, Jean-Michel Savoie

**Affiliations:** * INRA, UR1264 MycSA, Mycologie et Sécurité des Aliments, Villenave d’Ornon, France; † Departamento de Biologia, UFLA, Universidade Federal de Lavras, C.P. 3037, 37200-000, Lavras, MG, Brazil

**Keywords:** comparative mapping, macrosynteny, almond mushroom, button mushroom, chromosome evolution

## Abstract

Comparative linkage mapping can rapidly facilitate the transfer of genetic information from model species to orphan species. This macrosynteny analysis approach has been extensively used in plant species, but few example are available in fungi, and even fewer in mushroom crop species. Among the latter, the *Agaricus* genus comprises the most cultivable or potentially cultivable species. *Agaricus bisporus*, the button mushroom, is the model for edible and cultivable mushrooms. We have developed the first genetic linkage map for the basidiomycete *A. subrufescens*, an emerging mushroom crop known for its therapeutic properties and potential medicinal applications. The map includes 202 markers distributed over 16 linkage groups (LG), and covers a total length of 1701 cM, with an average marker spacing of 8.2 cM. Using 96 homologous loci, we also demonstrated the high level of macrosynteny with the genome of *A. bisporus*. The 13 main LG of *A. subrufescens* were syntenic to the 13 *A. bisporus* chromosomes. A disrupted synteny was observed for the three remaining *A. subrufescens* LG. Electronic mapping of a collection of *A. subrufescens* expressed sequence tags on *A. bisporus* genome showed that the homologous loci were evenly spread, with the exception of a few local hot or cold spots of homology. Our results were discussed in the light of *Agaricus* species evolution process. The map provides a framework for future genetic or genomic studies of the medicinal mushroom *A. subrufescens*.

The genus *Agaricus* includes several important cultivated mushroom crop species. Among those, *Agaricus bisporus* (Lange) *Imbach*, the button mushroom, is the most widely produced, and is consumed throughout the world. Given its agronomical importance, *A. bisporus* has been studied extensively at the genetic, molecular and physiological levels (Savoie *et al.* 2013). Genome sequence, molecular markers, linkage maps, QTL for various agronomical traits and breeding methods are available (Callac *et al.* 2006; Foulongne-Oriol *et al.* 2010, 2012a, 2012b; Gao *et al.* 2013, 2015; Morin *et al.* 2012), making *A. bisporus* the model organism for edible and cultivable basidiomycetes.

Lately, another *Agaricus* species, *Agaricus subrufescens* Peck, has received growing attention for its notable therapeutic properties. It is known to produce various bioactive compounds that have potential medicinal applications (Firenzuoli *et al.* 2008; Wisitrassameewong *et al.* 2012). Moreover, *A. subrufescens* can convert lignocellulosic residues into highly nutritious food, and in this way contribute to valorization of wastes. This basidiomycete, also known as the almond mushroom due to its particular flavor, has become, within a few years, one of the most important culinary-medicinal cultivable mushrooms, with potentially high added-value products and extended agronomical valorization (Largeteau *et al.* 2011; Llarena-Hernandez *et al.* 2013; Okuda *et al.* 2012). Today, a few commercial cultivars are available, all showing high genetic homogeneity (Colauto *et al.* 2002; Fukuda *et al.* 2003; Kerrigan 2005; Tomizawa *et al.* 2007). Breeding work on this species is in its infancy (Kerrigan and Wach 2008; Llarena-Hernandez *et al.* 2013) due to several bottlenecks, including a lack of knowledge on its ecology, reproductive biology, biodiversity, and genetics (Largeteau *et al.* 2011). The recent development of molecular markers and available expressed sequence tag (EST) resources have enriched our toolbox for studying the biology of this mushroom (Foulongne-Oriol *et al.* 2012c, 2014). The clarification of its amphithallic life cycle, and of the interfertility between field specimens from different continents (Rocha de Brito *et al.* 2016; Thongklang *et al.* 2014a), provide a sound basis to exploit the genetic diversity of this species, and to conduct controlled mating of selected genotypes.

The development of an attractive panel of cultivars of *A. subrufescens* is a challenging and long-term process. As a cultivable species, the traits of interest for selection are related to yield, quality, or disease control. Considering its medicinal properties, enrichment in bioactive compounds could also represent a target for selection. Most of those traits are under complex inheritance, making breeding much more arduous. Unraveling genetic control, in terms of gene numbers, effect, and genome position, is essential. Thus, as a prerequisite to develop such quantitative genetic approaches, the primary aim of this work was to develop a genetic linkage map of *A. subrufescens*. Markers with known homologs in *A. bisporus* (Foulongne-Oriol *et al.* 2014) used for the construction of the *A. subrufescens* map provide ground to explore the synteny between the two species. The conservation of chromosome-scale gene linkage relationships (macrosynteny), including, or not, the preserved order of the loci (collinearity) was assessed by a comparative mapping approach. Identifying commonalities and differences between the two *Agaricus* genome species will provide a basis to investigate evolutionary mechanisms and possible genomic divergence that have contributed to speciation process (Duran *et al.* 2009; Guyot *et al.* 2012). Besides phylogenetic considerations, comparative mapping enables the transfer of information between related species (Dirlewanger *et al.* 2004; Khazaei *et al.* 2014) and knowledge from the model species *A. bisporus* can benefit the orphan species *A. subrufescens*.

In the present study, we constructed the first genetic linkage map of *A. subrufescens*. By comparing the mapping position of *A. subrufescens* sequence tagged site markers with that of their homologs on the *A. bisporus* genome, we describe the macrosyntenic relationship between the two congeneric species. We interpret our results in the light of the species evolution process. Further possible applied outcomes for *A. subrufescens* breeding purposes are also discussed.

## Materials and Methods

### Mapping population

The parental hybrid strain CA487-100 × CA454-3 was previously obtained by crossing the two homokaryons CA487-100 and CA454-3, which are single spore isolates (SSIs) of the French and Brazilian strains CA487 and CA454 (a subculture of WC837), respectively (Llarena-Hernandez *et al.* 2013; Thongklang *et al.* 2014a). Single spores isolates were obtained from a spore print of the hybrid strain following the methodology described in Thongklang *et al.* (2014a). For each SSI, the level of ploidy (*n* + *n vs. n*) was determined with a multilocus genotype test based on 21 genomic microsatellite markers (hereinafter referred to as SubSSR for *A*. Sub*rufescens* simple sequence repeats) (Foulongne-Oriol *et al.* 2012c) that showed polymorphism between the two parental homokaryons: SSIs that did not exhibit heteromorphic profile at any loci were considered homokaryotic (Thongklang *et al.* 2014a). Among the 380 SSIs tested, 79 were found homokaryotic and were thus selected for further mapping purposes. The three parental strains (CA487-100, CA454-3, and the hybrid CA487-100 × CA454-3) and the haploid progeny of 79 homokaryons (referred hereafter as E*_i_*), are maintained in the “Collection du Germplasm des Agarics à Bordeaux” (CGAB), INRA-Bordeaux (http://www6.bordeaux-aquitaine.inra.fr/mycsa_eng/Biological-resources-of-value/The-Agaricus-culture-collection-CGAB).

### Genotyping

Total DNAs were extracted from freeze-dried mycelium following the protocol described in Zhao *et al.* (2011). DNA concentration was adjusted to 25 ng/µl, and the samples were stored at –20°.

The genotyping of genomic SubSSR markers (Foulongne-Oriol *et al.* 2012c) followed from the homokaryon selection test described above. In addition to this first set of 21 microsatellite markers, EST-SSRs (hereinafter referred to as ES-SSR) were also used for mapping (Foulongne-Oriol *et al.* 2014). Amplification conditions, electrophoretic separation, and visualization were performed according to Foulongne-Oriol *et al.* (2012c). All microsatellite primers used for mapping are listed in Supplemental Material, Table A in File S1.

Sequence-tagged site (STS) markers were developed on the basis of sequences available from the literature (Foulongne-Oriol *et al.* 2012c, 2014; Matsumoto-Akanuma *et al.* 2006; Thongklang *et al.* 2014a). The cleaved amplified polymorphic sequence (CAPS) approach was used to reveal polymorphism in STS. PCR primer pairs were designed to amplify products between 300 and 500 bp using Primer 3 software with default parameters (Rozen and Skaletsky 2000). Amplifications, digestion, electrophoretic separation, and visualization were performed according to Foulongne-Oriol *et al.* (2010). For each STS marker (hereinafter referred to as PRS), primers and corresponding restriction enzymes are described in Table A in File S1.

Amplified fragment length polymorphism (AFLP) markers were genotyped using the settings described by Foulongne-Oriol *et al.* (2010). Total DNA was digested with the *Eco*RI and *Mse*I endonucleases. *Eco*RI and *Mse*I primers with, for both, two selective nucleotides, were used for amplification through seven pair combinations (Table B in File S1). The amplified fragments were separated and visualized on an ABI3130 sequencer (Applied Biosystems). Electropherograms were analyzed with GENEMAPPER V4.0 software. For each primer pair, the reference panel used for allele calling in the progeny was based on the AFLP patterns of the three parental strains (presence/absence in one of the two homokaryons, presence in the hybrid).

The allelic segregation at the mating-type locus (*MAT*) was determined by pairing each homokaryon E*_i_* h with the two homokaryons CA454-3 (*MAT-2*), and CA487-100 (*MAT-4*). The *MAT* allele of each E*i* was deduced from unambiguous compatible reaction with one tester or the other. Mating tests were performed as described in Thongklang *et al.* (2014a) with two replicates per confrontation. Thus, the allele at the *MAT* locus could be determined for 74 E*_i_* (93.6%).

### Linkage map construction

Genotypic data were independently scored by two experimenters to minimize scoring errors. Locus segregation was tested for deviation from expected Mendelian ratios 1:1 with a *χ*^2^ test. Linkage and mapping analysis was performed using Mapmaker/exp V3.0b software (Lander *et al.* 1987). The recombination frequency was converted into Kosambi centimorgan (cM) units. In a first round of mapping, markers having more than 30% of missing data, and/or markers with a skewed segregation ratio (*P* < 0.05), were omitted to limit spurious linkage. AFLP markers were kept out of this first step, and those with unbalanced allelic segregation were definitely removed from the genotyping data set. A minimum LOD score of 4.0 and maximum distance of 30 cM were set as thresholds for linkage groups (LG) determination with the ‘group’ command. For each group, the most likely marker order was established using the ‘order’ command. Marker orders were confirmed with the ‘ripple’ command. A framework map was thus established. In a second step, markers excluded from the first round of mapping were tested for linkage using the ‘group’ and ‘links any’ commands. The ‘assign’ command (LOD > 6) allowed the assignation of these markers to the LG defined in the first step. For each LG, additional markers were sequentially placed using the ‘try’ commands. Markers that were difficult to place with several likely positions were dropped. Subsequent orders were again tested with the ‘ripple’ command.

MAPCHART software (Voorrips 2002) was used for graphical representation of the linkage map. Linkage groups were numbered in descending order based on their length in centimorgans. The occurrence of crossovers for each LG and each individual was analyzed using Graphical Genotyping (GGT 2.0) (van Berloo 2008). Estimated genome length *L_e_* was determined from the linkage data according to Hulbert *et al.* (1988), as modified by Chakravarti (Chakravarti *et al.* 1991, Method 3). Map coverage was calculated using the formula *P* = 1 − (1 − 2*c*/*L_e_*)*^m^*, where *P* is the proportion of the genome within 2*c* cM of a marker, *m* is the number of informative markers on the map, and *L_e_* is the estimated genome length (Beckmann and Soller 1983).

### Assessment of A. subrufescens–A. bisporus macrosynteny

All the sequence-based markers used for the presented linkage map construction were used to infer syntenic relationships between *A. subrufescens* and *A. bisporus* chromosomes.

In a previous study, *A. subrufescens* EST sequences were aligned on the genome sequence of *A. bisporus* (Foulongne-Oriol *et al.* 2014). As a brief reminder, the 10,114 *A. subrufescens* sequences were mapped to *A. bisporus* scaffolds (H97 sequence V2.0, http://genome.jgi-psf.org/Agabi_varbisH97_2/Agabi_varbisH97_2.home.html; Morin *et al.* 2012) using the BLAT algorithm (Kent 2002). A score of 60%, which corresponds to the product of the first quartile value for alignments coverage (75%) and identity (80%), was used as threshold to filter out unreliable results. Putative *A. bisporus* genes homologous to *A. subrufescens* ESTs were searched for by comparing best hit mapping coordinates to *A. bisporus* gene model (GM) coordinates. Thus, 6752 *A. subrufescens* sequences were mapped on the *A. bisporus* genome, among which 6570 overlapped with 3620 *A. bisporus* GM (34.93% of all GM) (Foulongne-Oriol *et al.* 2014). To go further in the present study, *A. bisporus* homolog distribution along the genome was examined per scaffold, and, on each scaffold, per window of 50 kb. For the others markers, significant homology with *A. bisporus* gene was inferred by blastn search similarity (e-value < 1 × 10e^–10^).

The syntenic relationships established between *A. subrufescens* linkage map and *A. bisporus* chromosome sequences were visualized using CIRCOS (Krzywinski *et al.* 2009). The level of collinearity between genetic and physical orders was assessed by comparing, between map and genome, the coordinate and orientation of each homologous pairs’ intervals.

### Data availability

Strains are available upon request. Information about markers used or developed specifically for this project can be found in Table A and Table B of File S1. The genotype dataset used to build the genetic map is available in Table S1. The *A. subrufescens* EST sequences are accessioned in GenBank with the reference GBEJ00000000.1. Coordinate data resulting from the alignment of these *A. subrufescens* EST sequences on the *A. bisporus* genome are available in Foulongne-Oriol *et al.* 2014 or upon request.

## Results

### Genotyping, segregation and linkage analysis, and map construction

In addition to the 21 SubSSR markers used for homokaryon selection, 18 ES-SSR found polymorphic between the two parental strains were used for mapping. Ninety-four polymorphic CAPS markers were developed from available *A. subrufescens* sequences. The AFLP technique was used in order to increase mapping coverage. A total of 90 polymorphic and reliable AFLP markers were identified using seven primer pairs.

A set of 224 markers (90 AFLPs, 39 SSRs, 94 CAPSs, and the *MAT* locus) was analyzed for mapping purposes. Twenty-eight markers (12.5%) showed a segregation ratio that deviated from the expected 1:1 ratio (*P* < 0.05). The total number of genotyped individuals per locus varied from 45 to 79, with an average of 72. Nearly 97% of the scored markers showed less than 20% of missing data.

The first round of mapping included 106 markers representing the most confident and informative set of genotyped loci (only unbiased codominant markers with less than one-third of missing data). With our mapping condition (LOD > 4, *d*_max_ < 30 cM), a preliminary framework map was established with 97 markers distributed over 18 LG. The next rounds of mapping consisted in placing the remaining markers. The addition of markers allowed the merging of four groups into two larger ones. For the other groups, the sequential mapping procedure led to an increase of marker density and length. Twenty-two markers (14 AFLPs, two SSRs, and six CAPSs) were discarded from the current mapping dataset due to inconsistent localization or substantial increases of map length.

### Map features

**Figure 1:**
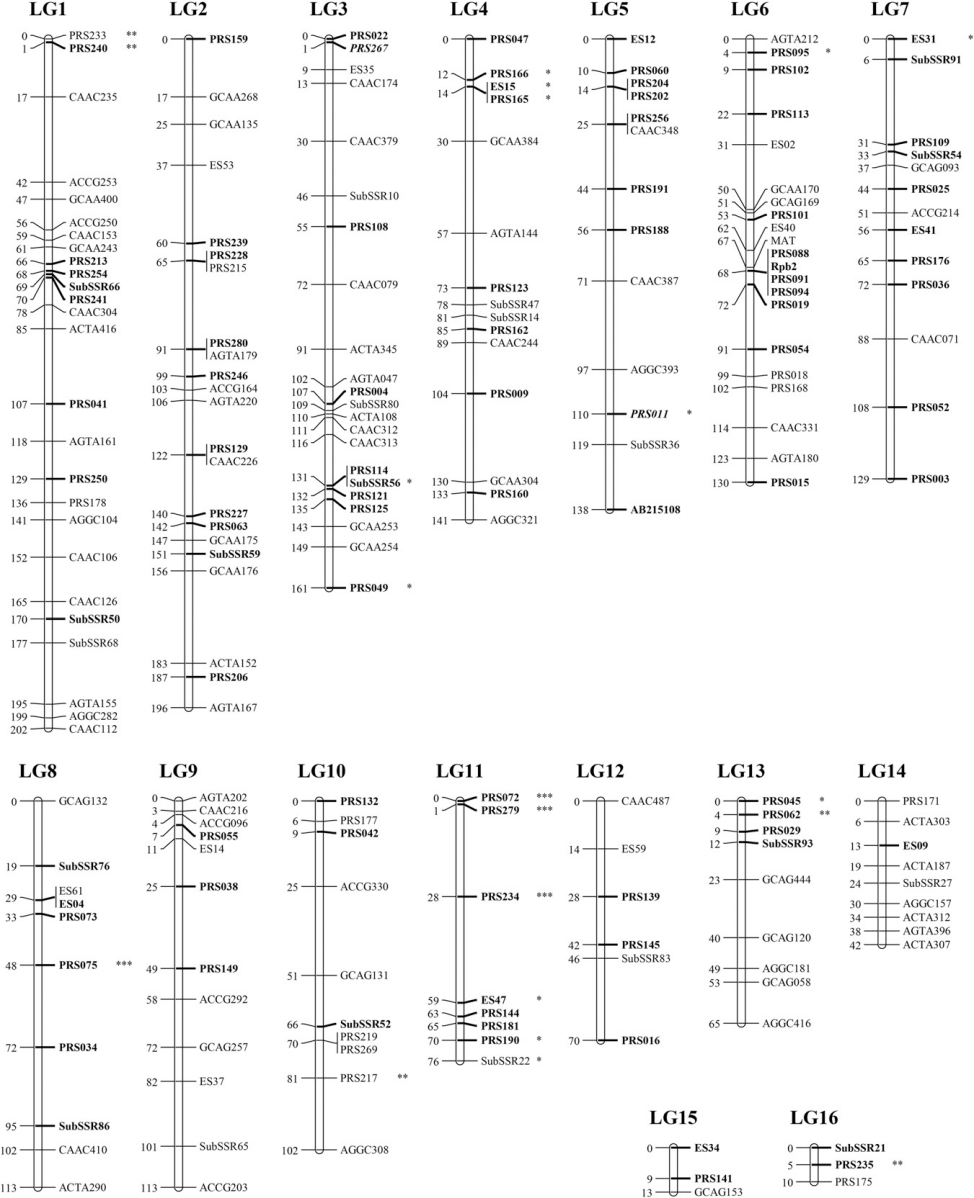
Genetic linkage map of *A. subrufescens*. Markers with homologs found in *A. bisporus* genome are in bold. Italicized markers show inconsistent mapping location according to the physical position of their homologs. Markers exhibiting significant segregation distortion are indicated with asterisk (* *P* < 0.05, ** *P* < 0.01, *** *P* < 0.001).

The final *A. subrufescens* linkage map includes 202 markers (76 AFLPs, 37 SSRs, 88 CAPS, and the *MAT* locus; segregating data are available in Table S1) distributed over 16 LG, and covers a total length of 1701 cM, with an average distance between adjacent markers of 8.2 cM (Table 1 and Figure 1). According to their number of constituent markers and their relative length, 14 major LGs (from 1 to 14), built with more than six markers (length > 40 cM), and two minors ones (15, 16), based on only three linked markers (length < 15 cM) can be distinguished (Table 1). Most of the intervals between two adjacent markers (89.7%) are less than 20 cM (60.3% in less than 10 cM), and the largest interval is of 30 cM (between ES47 and PRS234 on LG11). Considering the major LGs, the mean crossover frequency per LG per individual varies from 0.39 (LG 14) to 2.06 (LG 1), with an average of 1.13, and was highly correlated with the number of markers (*r* = 0.90, *P* = 8.6 × 10^−6^). Of the 202 markers used for map construction, 21 (10.4%) show significant segregation deviation from the expected 1:1 ratio (*P* < 0.05) (Table S1). One-third concerns isolated markers, suggesting that the observed bias could be due to possible genotyping scoring errors. The other markers showing skewed segregation are not found randomly distributed, but tend to cluster on distal position of linkage groups (Figure 1). The genetic segments formed by successive biased markers represent less than 4.3% of the linkage map. Linkage group 11 alone exhibits a large proportion of markers with unbalanced segregation (6/8). Of the 21 mapped markers with skewed segregation, 19 have an excess of CA487-100 parental strain alleles. The only two markers showing a bias toward an excess of CA454-3 allele are PRS217 (LG10) and ES31 (LG7).

**Table 1:** Characteristics of the *A. subrufescens* genetic linkage groups

	Linkage Group	Length (cM)	No. of Markers	No. of Distorted Markers*^a^*	Average Marker Spacing (cM)	Largest Interval (cM)
	1	202	26	2	7.8	23
	2	196	22	0	8.9	27
	3	161	22	2	7.3	19
	4	141	15	3	9.4	27
	5	138	13	1	10.6	26
	6	130	21	1	6.2	19
	7	129	13	1	9.9	24
	8	113	10	1	11.3	24
	9	113	12	0	9.4	24
	10	102	10	1	10.2	26
	11	76	8	6	9.5	30
	12	70	6	0	11.7	26
	13	65	9	2	7.2	17
	14	42	9	0	4.7	6
	15	13	3	0	4.3	9
	16	10	3	1	3.3	5
Total	16	1701	202	21	—	—
Mean	—	106.3	12.6	1.3	8.2	21

a Markers showing unbalanced segregation ratios (*P* < 0.05).

The estimated genome length *L_e_* was 2458 cM, expanding the observed map length of 44.5%. According to this estimate, the proportion of the genome within 20 cM of a marker is 79%. To reach 95% of saturation with the same mapping precision, the number of markers should be increased 2.3-fold.

### Synteny with the A. bisporus genome

**Figure 2:**
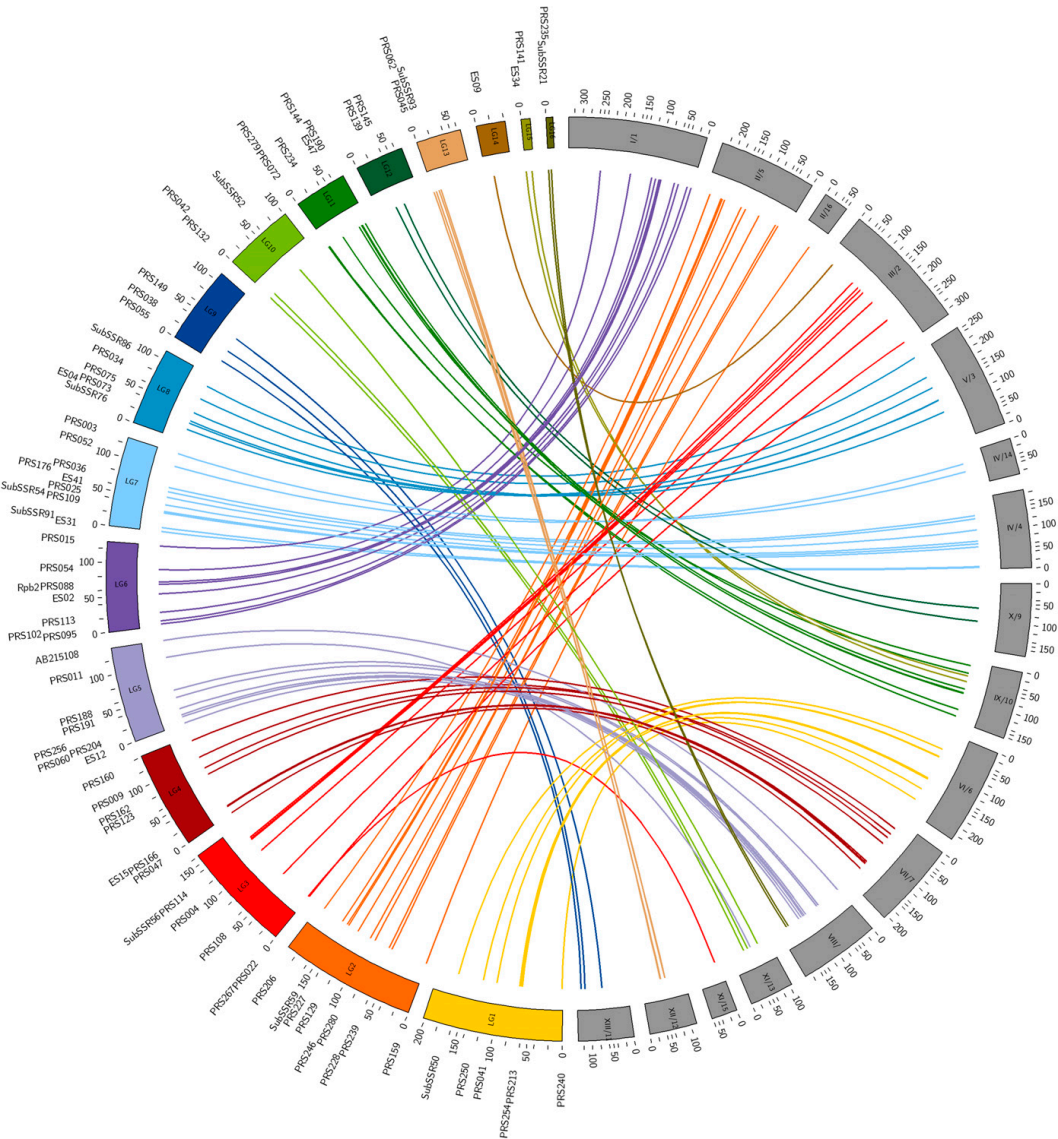
Graphical representation of syntenic relationship between *A. subrufescens* linkage map and *A. bisporus* genome. Linkage groups (LG) of *A. subrufescens* are depicted in colors. The chromosomes and the corresponding scaffolds (chr/scaff) of *A. bisporus* (Morin *et al.* 2012) are in gray, and have been reordered to minimize lines overlapping. Lines of the same color as the *A. subrufescens* LG connect genetic molecular markers with the physical position of their homologs on *A. bisporus* genome. The units of the LG are given in centimorgan for *A. subrufescens* map and 10^4^ bases for *A. bisporus* genome.

Among the 125 *A. subrufescens* (As) sequence-based markers, 96 showed similarities to *A. bisporus* genes (Ab) (Table 2). The map location of these markers, and the physical position of their corresponding homologs in the genome of *A. bisporus* were compared. In most cases (98%), the markers genetically linked in *A. subrufescens* are physically related in *A. bisporus* (Table 2, Figure 2, and Figure A in File S1) and bound syntenic blocks. Two exceptions are observed for PRS267 and PRS011. These two markers, mapped on As-LG3 and As-LG5, respectively, have their homologs (Ab genes ID 1982365 and 195996) physically assigned to the Ab-chromXI instead of Ab-chromIII, and Ab-chromVIII as expected regarding the other adjacent mapped loci. The marker PRS011 derives from an As-EST sequence that shows strong homology with a gene encoding a cytochrome P450 monooxygenase. Therefore, the development of a locus-specific marker from sequence belonging to such a multigenic family could be quite hazardous. This suspicion was confirmed by the results of electronic mapping, since several hits on various scaffolds were returned. Among them, an equally likely hit (e-value < 1 × 10e^–17^) was found on scaffold 8 (Ab-chromVIII), consistent with the genetic location. For PRS267, a unique homolog (gene ID 212249) was found on Ab-chromIX. However, this gene is annotated as encoding for a hypothetical protein containing an abhydrolase_3 domain. In the genome of *A. bisporus* (Morin *et al.* 2012), six other genes with the same functional domain encode putatively proteins of the alpha/beta hydrolase superfamily, but none of them is located on Ab-chromIII. A local chromosomal rearrangement could not be excluded to explain the position of PRS267. The other As-markers without Ab-homologs are spread evenly over the As LG.

**Table 2:** Oxford grid showing conservation of synteny between *A. subrufescens* linkage groups (rows) and *A. bisporus* chromosomes (columns), sorted by the As-LG number

		*A. bisporus*													
	LG\chrom.	VI	II	III	VII	VIII	I	IV	V	XIII	XI	IX	X	XII	Total
*A. subrufescens*	1	8	0	0	0	0	0	0	0	0	0	0	0	0	8
	2	0	10	0	0	0	0	0	0	0	0	0	0	0	10
	3	0	0	8	0	0	0	0	0	0	*1*	0	0	0	9
	4	0	0	0	8	0	0	0	0	0	0	0	0	0	8
	5	0	0	0	0	8	0	0	0	0	*1*	0	0	0	9
	6	0	0	0	0	0	11	0	0	0	0	0	0	0	11
	7	0	0	0	0	0	0	10	0	0	0	0	0	0	10
	8	0	0	0	0	0	0	0	6	0	0	0	0	0	6
	9	0	0	0	0	0	0	0	0	3	0	0	0	0	3
	10	0	0	0	0	0	0	0	0	0	3	0	0	0	3
	11	0	0	0	0	0	0	0	0	0	0	7	0	0	7
	12	0	0	0	0	0	0	0	0	0	0	0	3	0	3
	13	0	0	0	0	0	0	0	0	0	0	0	0	4	4
	14	0	0	1	0	0	0	0	0	0	0	0	0	0	1
	15	0	0	0	0	0	0	0	0	0	0	2	0	0	2
	16	0	0	0	0	2	0	0	0	0	0	0	0	0	2
	Total	8	10	9	8	10	11	10	6	3	5	9	3	4	96

Each number in cell denotes the number of homologous pair of loci.

The alignment of As marker–Ab homolog pairs along LG and scaffolds reveals an extensive macrosyntenic relationship (Table 2, Figure 2, and Figure A in File S1), together with a strong collinearity. Between one (As-LG14/Ab-chromVI) and 11 (As-LG6/Ab-chromI) pairs of As-marker/Ab-homologs per LG, with an average of six, allowed the analysis of synteny. Indeed, nearly 80% of the *A. subrufescens* linkage map can be related to the genome of *A. bisporus*. In most cases, synteny blocks span whole As-LG and enables the inference of homologous chromosomes between *A. subrufescens* and *A. bisporus*. The main As LG are found syntenic to the 13 Ab chromosomes. The As-LG14 and the As-LG3 are both found syntenic with the Ab-chromIII. The As-LG14 is anchored on the distal part of the chromosome (Figure 2 and Figure A in File S1). The same pattern of synteny is also observed between As-LG5/As-LG16 and Ab-chromVIII, the corresponding Ab-homologs of As-LG16 markers being located on one end of the chromosome. For these two cases of synteny discrepancies, the LG under consideration are found linked at mapping thresholds less stringent (LOD > 3, *d*_max_ < 35 cM). Regarding the synteny between As-LG11 and As LG15 with Ab-chromIX, the Ab-homologs of the markers mapped to LG15 were found flanked on each side by Ab-homologs of As-LG11 markers (Figure 2). A most permissive mapping procedure did not allow a genetic linkage to be surmised between these LG. The collinearity is mostly respected, with the genetic order of the mapped markers being consistent with the physical order of their homologs (Figure 2 and Figure A in File S1). Indeed, 77.3% of the marker pairs’ intervals were found collinear with their homologous ones. Some local inversions are observed on LG1, LG2, LG3, LG4 or LG12. The pattern observed on LG8 suggests an inversion of the genetic interval comprising between SubSSR76 and PRS075. Several breakdown of collinearity were observed on LG11.

**Figure 3:**
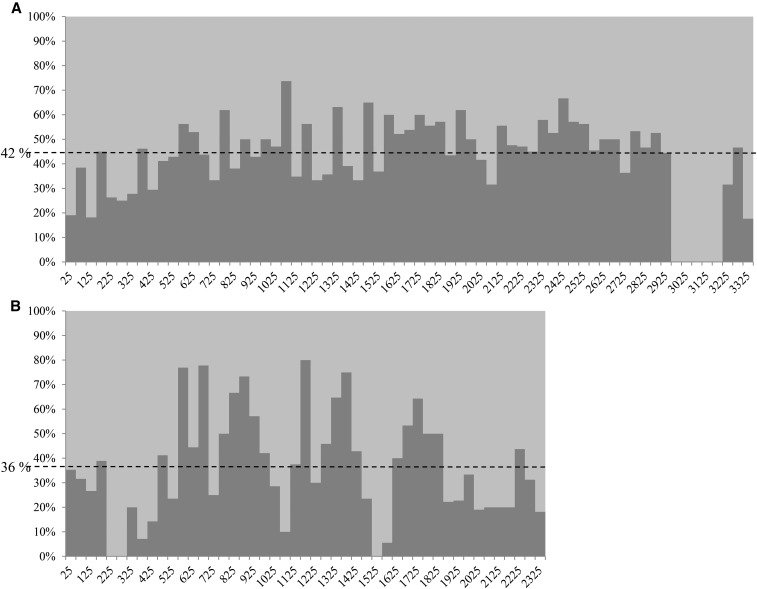
Distribution of the percentage of overlapped *A. bisporus* gene models (GM) (dark gray) by *A. subrufescens* sequence per 50 kb-window on chromosome I (scaffold 1) (A) and chromosome VI (scaffold 6) (B), piled at 100% of GM. The dotted line is the average percentage of overlapped *A. bisporus* GM for each chromosome.

Alignment of the *A. subrufescens* EST sequences on the *A. bisporus* genome showed that hits to homologous loci were found on all the main scaffolds (Table 3). The average distance between any two electronically mapped sequences based on their *A. bisporus* homologs locations was 7.2 kb. The number of GM overlapped by *A. subrufescens* sequences per chromosome was found to be highly correlated with the total number of GM per chromosome (*r* = 0.97, *P* = 5.35 × 10e^–13^). Taken as a whole, the distribution per 50 kb of overlapped GM along the chromosomes is consistent with the GM distribution within the same interval. For illustration, the distribution of overlapped GM on chromosome I (scaffold 1) follows the distribution of the GM (Figure 3A), with the lowest density of overlapped GM observed between 2.9 Mb and 3.2 Mb, which corresponded to the lowest density in GM due to the presence of repeated elements. However, an unexpected pattern of homolog distribution was observed for some chromosomes (V, VI, VIII, IX, and XI) (*χ*^2^ test, *P* < 0.01), with hot spots (higher number of homologs than expected), and/or cold spots (lower number than expected), of homology. These distinct regions were found distributed randomly on the linkage group, as illustrated for chromosome VI on Figure 3B. Beyond cold spots of homology related to repeats region (around 0.22 Mb and around 1.52 Mb), other genomic segments exhibit either fewer (1075–1125 kb) or more (1175 –1225 kb) homologs.

**Table 3:** Results of *A. subrufescens* EST sequence similarity searches against *A. bisporus* gene models

	*A. bisporus* Chromosome	Scaffold on *A. bisporus* Genome V2.0	No. of *A. subrufescens* Mapped Sequences	No. of *A. subrufescens* Sequences Overlapping *A. bisporus* GM	No. of *A. bisporus* GM Overlapped	% of *A. bisporus* GM Overlapped
	I	scaffold 01	1065	1038	540	43.34
	III	scaffold 02	842	828	449	40.20
	V	scaffold 03	495	477	280	31.96
	II, IV	scaffold 04	638	621	340	37.74
	II	scaffold 05	659	638	364	41.65
	VI	scaffold 06	558	540	285	36.26
	VII	scaffold 07	485	476	259	36.79
	VIII	scaffold 08	258	251	145	23.81
	X	scaffold 09	289	270	153	26.33
	IX	scaffold 10	313	308	172	32.09
	XIII	scaffold 11	229	228	127	30.98
	XII	scaffold 12	191	184	106	30.11
	XI	scaffold 13	170	162	93	27.43
	IV	scaffold 14	220	217	121	38.91
	XI	scaffold 15	105	104	57	27.27
	II	scaffold 16	97	95	48	25.40
	XII	scaffold 17	67	63	44	25.43
	IV	scaffold 18	33	33	22	28.21
	VII	scaffold 19	34	33	13	21.31
	ND	scaffolds 22, 29	4	4	2	17.15
Total			6752	6570	3620	—
Mean			337.60	328.50	181.00	31.12

## Discussion

The construction of genetic linkage maps in cultivable mushrooms has been restricted mostly to economically important agaricomycete species such as *A. bisporus* (Foulongne-Oriol *et al.* 2010, 2011), *Pleurotus* spp. (Larraya *et al.* 2000; Okuda *et al.* 2012), and *Lentinula edodes* (Gong *et al.* 2014; Kwan and Xu 2002). In this study, we present the first genetic linkage map for the medicinal mushroom *A. subrufrescens* using sequence tag site and AFLP markers. The availability of such a molecular tool constitutes a milestone for further genetic studies in this species. The near-complete map presented is a solid backbone of the *A. subrufescens* genome, and makes possible the initiation of QTL analyses. The dissection of complex inherited traits of economic importance, such as yield components, can be done (Foulongne-Oriol 2012). In the particular case of this medicinal mushroom, the identification of QTL controlling the production of bioactive compounds can contribute to unraveling their biosynthetic pathways, as well as their biological functions in the mushroom. This approach was undertaken successfully to identify the genetic basis of biotechnological added-value metabolites variations in yeast (Breunig *et al.* 2014), and in *P. ostreatus* (Santoyo *et al.* 2008). Further applications, such as fine mapping or map-based cloning, can also be considered. In this way, the use of nonanonymous markers would facilitate subsequent resolution of mapped genomic regions to candidate genes. The availability of *A. subrufescens* EST catalogs as a source of molecular markers will greatly enhance the development of targeted loci related to specific pathway or traits of interest. Our results confirm also that the recombination events occur normally in *A. subrufescens*, as already suggested (Thongklang *et al.* 2014a; Rocha da Brito *et al.* 2016). Such recombination ability is of particular interest for mushroom breeding purposes since it will facilitate the creation of favorable allelic combinations in breeding schemes.

Syntenic relationships have been studied widely in plant and animal species, but until recently such comparisons have been performed only rarely in fungi. The availability of complete fungal genome sequences has made the analysis of synteny between genomes possible through comparative genomics studies (Thon *et al.* 2006; Hane *et al.* 2011; Ohm *et al.* 2012). In our case, the lack of a reference genome sequence for *A. subrufescens* did not allow such an approach. Therefore, the construction of the *A. subrufescens* linkage map affords an opportunity to explore the syntenic relationship with the species *A. bisporus* thanks to a set of 96 homologous loci. In this way, we demonstrate the extensive conservation of synteny and gene order between *A. bisporus* and *A. subrufescens*, suggesting that the genome structures of these two species have not diverged drastically. Such a comparative mapping strategy has been applied only rarely in fungi between congeneric species. For example, De Vos *et al.* (2014) demonstrated genome-wide macrosynteny, with a few chromosome rearrangements, among the *Gibberella fujikoroi* species complex. The present study is the first devoted to synteny analysis between mushroom species. For each As LG, homology with the Ab chromosome could be inferred, but one-to-one correspondence was not fully established. The 13 main As linkage groups were found syntenic to the 13 Ab chromosomes, but the situation was not clear for the three remaining As linkage groups with an observed disrupted synteny relationship. In this case, increasing marker density from an additional mapping effort could answer the question of whether there is a linkage gap between two parts of a unique chromosome, or if there are several segregating chromosomes. The number of haploid chromosomes in *A. subrufescens* remains unknown to date, but our results suggest that this number is greater than, or equal to, 13. The two *Agaricus* sections *Arvenses* and *Bivelares* to which *A. subrufescens* and *A. bisporus*, respectively, belong, have diverged from their common ancestor approximately 30 million years ago (MYA) according to Lebel and Syme (2012). Chromosome rearrangements are likely to occur during speciation processes, and represent an important factor in fungal genome evolution (Giraud *et al.* 2008). In *A. bisporus*, the location of a unique putative centromere per chromosome (Foulongne-Oriol *et al.* 2010), and the absence of interstitial trace of remnant of telomere (Foulongne-Oriol *et al.* 2013), suggest that the 13 chromosomes of the species did not resulted from fusion. Thus, if the ancestral chromosome number is 13, a possible fission of chromosomes could explain the macrosyntenic pattern observed between *A. subrufescens* and *A. bisporus*. Cytological chromosome counts and electrophoretic karyotyping would ascertain the haploid number of chromosomes in *A. subrufescens*. In the light of our results, the overall gene collinearity between the two species was evidenced, but local structural changes could not be completely excluded. Possible rearrangement has been already suggested in a previous mapping study based on another progeny analysis to explain the loss of synteny for the rDNA (ITS) locus chromosome assignment between the two species (Rocha de Brito *et al.* 2016).

To go further, the high level of synteny between Ab-chromosome I and As-LG6 deserves particular attention. The *A. bisporus* chromosome I carries the *MAT* locus, located in the pericentromeric region and governing sexual compatibility (Xu *et al.* 1993). Thongklang *et al.* (2014a) showed that *A. subrufescens*, like *A. bisporus*, has a unifactorial system of sexual incompatibility with a *MAT* locus. In the present study, we localized the As-*MAT* locus on As-LG6, homologous to Ab-chromosome I. This result suggests a possible functional conservation of the locus. A deeper analysis of the microsynteny at the sequence level could confirm such an observation. The tight genetic linkage between the As-*MAT* locus and the marker PRS091, which is derived from the putative *A. subrufescens mip* gene (Thongklang *et al.* 2014a) known to flank the *MAT* locus (James *et al.* 2004), represents the first evidence in this direction.

Beyond evolutive considerations, consequences of the observed conserved macrosynteny between these two agronomic mushroom species can be discussed in the light of applied issues. An analogy between *A. bisporus* and *A. subrufescens* can be drawn. The cultivation process of *A. subrufescens* has been modeled mainly on the methods used to grow *A. bisporus*. Both are secondary decomposers, and can be cultivated on similar substrates, but *A. subrusfescens* requires higher temperature conditions for fruiting. Others agronomic parallels between the two cultivated species can be pointed out: similar yield components, common biotic disorders, comparable postharvest issues (Largeteau *et al.* 2011; Llarena-Hernandez *et al.* 2013, 2014). The high level of synteny demonstrated between the two species renders the possible transfer of knowledge gained on *A. bisporus* to *A. subrufescens* conceivable. We may therefore expect that QTL controlling traits such as yield would be found conserved between the two species. Such comparative QTL mapping studies have been done successfully in various plants (Chagné *et al.* 2003; Li *et al.* 2013), and our results provide the first evidence that this approach could also be applied to fungal species. Since we have demonstrated that *A. subrufescens* EST with known *A. bisporus* homologs are well distributed on the *A. bisporus* genome, these sequences provides a sound basis from which to perform further synteny-based genomic studies. Besides applications in genetics and breeding, comparative QTL mapping could be seen as a tool to predict regions of homology, and could provide information on functional synteny.

To conclude, we have constructed the first genetic linkage map of *A. subrufescens*, providing a framework for the future genetic studies of this medicinal species. This map, based on sequence-tagged site markers, will be very useful for prospective *A. subrufescens* genome sequencing projects. We have proved, through a comparative mapping approach with the *A. bisporus* genome, a high degree of synteny conservation between the two species. Since *Agaricus* is potentially the most cultivable mushroom genus, our results are also promising for other species such as *A. bitorquis*, *A. arvensis*, or *A. flocculosipes* (Thawthong *et al.* 2014; Thongklang *et al.* 2014b). In addition, we have also demonstrated that the *A. subrufescens* EST sequences provide a potential source of syntenic markers that could be custom-made to target specific genomic regions. Beyond macrosynteny, we may imagine a potential transferability of the genetic information between the two species. We have found the first evidence of possible functional conservation between the two species with the location of the *MAT* locus on homolog chromosomes. The present work provides landmarks to guide further exploration of the evolutionary story of these two species, and their process of speciation.

## Supplementary Material

Supplemental Material
