# Supplementary material for: The Genetic Linkage Map of the Medicinal Mushroom Agaricus subrufescens Reveals Highly Conserved Macrosynteny with the Congeneric Species Agaricus bisporus
Source: G3 (Bethesda). 2016 Feb 26;6(5):1217–26. doi: 10.1534/g3.115.025718 (PMC4856074; doi:10.1534/g3.115.025718)
Supplement: Supplemental Material [file supp_6_5_1217__index.html]

The Genetic Linkage Map of the Medicinal Mushroom Agaricus subrufescens Reveals Highly Conserved Macrosynteny with the Congeneric Species Agaricus bisporus — Supplemental Material 

# The Genetic Linkage Map of the Medicinal Mushroom *Agaricus subrufescens* Reveals Highly Conserved Macrosynteny with the Congeneric Species *Agaricus bisporus*

## Supplemental Material for Foulongne-Oriol *et al.*, 2016

**Files in this Data Supplement:**

- File S1 - File contains: Table A. Characteristics of SSR and CAPS markers used for the construction of the genetic linkage map of *Agaricus subrufescens*. Table B. The 7 AFLP primers combinations used for the genotyping of *A. subrufescens* progeny. Figure A. Graphical representation of syntenic relationships between *A. subrufescens* linkage map and *A. bisporus* genome. (.pdf, 506 KB)
- Table S1 - Marker data matrix. (.xlsx, 91 KB)
