# Supplementary material for: The Genetic Linkage Map of the Medicinal Mushroom Agaricus subrufescens Reveals Highly Conserved Macrosynteny with the Congeneric Species Agaricus bisporus
Source: G3 (Bethesda). 2016 Feb 26;6(5):1217–26. doi: 10.1534/g3.115.025718 (PMC4856074; doi:10.1534/g3.115.025718)
Supplement: Supplemental Material [file supp_g3.115.025718_FileS1.pdf]

The genetic linkage map of the medicinal mushroom *Agaricus subrufescens* reveals highly conserved macrosynteny with the congeneric species *Agaricus bisporus*

Marie Foulongne-Oriol[1]*, Manuela Rocha de Brito[1,2], Delphine Cabannes[1], Aurélien Clément[1], Cathy Spataro[1], Magalie Moinard[1], Eustáquio Souza Dias[2], Philippe Callac[1], Jean-Michel Savoie[1]

[1] INRA, UR1264 MycSA, Mycologie et Sécurité des Aliments, Villenave d'Ornon, France
[2] UFLA, Departamento de Biologia, Universidade Federal de Lavras, C.P. 3037, 37200-000, Lavras, MG, Brazil

*Corresponding author.

E-mail: mfoulong@bordeaux.inra.fr

G3: Genes/Genomes/Genetics

Supplementary Material: File S1. Table A, Table B, Figure A

<u>Table A</u>: Characteristics of SSR and CAPS markers used for the construction of the genetic linkage map of *Agaricus subrufescens* . In bold are indicated the homologous loci used for comparative mapping.

Locus name	LG on *A. subrufescens* map	Forward primer	Reverse Primer	Endonuclease	reference for primer design	designed from	reference for the sequence	Mapping on *A. bisporus* H97 V2.0	Chromosome *A. bisporus*	Mapping-start	Mapping-end	Homolog gene ID in *A. bisporus* genome
**AB215108**	5	GTTCAAGCCCTCGGTAATTTCT	CCAATCCCAGTATGGGTGAC	*Mse* I	this study	AB215108	Matsumoto-Akanuma et al. 2006	scaffold_8	VIII	21924	22242	239418
**Cel4**	2	GCTTGTGTCGAACAGAACGA	GGCAACTATCAAGCGAATCC	*Hpy* CH4V	Thongklang et al. 2014	KJ541824	Thongklang et al. 2014	scaffold_5	II	1202978	1203601	192609
ES02	6	TGAAAAATCTAACCCCAGCC	TGTCCCAGAAGCACACTCTC	-	Foulongne-Oriol et al. 2014	GBEJ01009376	Foulongne-Oriol et al. 2014	unassigned	-	-	-	-
**ES04**	8	CCTTGCCCTAAATTACCCAA	CCCTCTTCCTCTTCCTCCTT	-	Foulongne-Oriol et al. 2014	GBEJ01009391	Foulongne-Oriol et al. 2014	scaffold_3	V	1172835	1174090	191238
**ES09**	14	TTCAGATGCTGCCCTTACTG	GGCAAAGACTGTGATGATGG	-	Foulongne-Oriol et al. 2014	GBEJ01006996	Foulongne-Oriol et al. 2014	scaffold_2	III	202867	203392	114802
**ES12**	5	AGCCGCAAAGGACTACAACT	AGAAAAGCCCAGACAAGGAA	-	Foulongne-Oriol et al. 2014	GBEJ01008518	Foulongne-Oriol et al. 2014	scaffold_8	VIII	1325040	1326846	194237
ES14	9	CCATGATGCCTTTGAGTGAC	GAAGACGAACTCGACGACAA	-	Foulongne-Oriol et al. 2014	GBEJ01008548	Foulongne-Oriol et al. 2014	unassigned	-	-	-	-
**ES15**	4	AAAATCAACGAAGTCCTGGG	GGCTGATGAGAAGAAGGAGG	-	Foulongne-Oriol et al. 2014	GBEJ01008204	Foulongne-Oriol et al. 2014	scaffold_7	VII	1400101	1405428	207384
**ES31**	7	TTCTTGGGATGTGATGTTGC	GACCACTTCCCCAAAAGAAA	-	Foulongne-Oriol et al. 2014	GBEJ01000928	Foulongne-Oriol et al. 2014	scaffold_4	IV	41407	42000	191595
**ES34**	15	TGGATGGCTGAGTCGTAGTC	AAGCCAGGTGGAATATTTGG	-	Foulongne-Oriol et al. 2014	GBEJ01001278	Foulongne-Oriol et al. 2014	scaffold_10	IX	393478	393981	226482
ES35	3	GGAAAGGTGTTAAAACGGGA	AATATCAAATGTCCCGGCTC	-	Foulongne-Oriol et al. 2014	GBEJ01001328	Foulongne-Oriol et al. 2014	unassigned	-	-	-	-
ES37	9	CTGTGGATGATGATCCTGCT	GACGGTAAAATCCCGAGAAA	-	Foulongne-Oriol et al. 2014	GBEJ01002594	Foulongne-Oriol et al. 2014	unassigned	-	-	-	-
ES40	6	AGTGGTTAAGTGAGGTGGGG	AGATGCCCAGAACATGTCAA	-	Foulongne-Oriol et al. 2014	GBEJ01002813	Foulongne-Oriol et al. 2014	unassigned	-	-	-	-
**ES41**	7	TACAAAACAGGGACCGAACA	CACCTGTTGATTTCAGTGGG	-	Foulongne-Oriol et al. 2014	GBEJ01007280	Foulongne-Oriol et al. 2014	scaffold_4	IV	1047656	1048451	191881
**ES47**	11	TCGTTGAGCAAGATTGGAAG	GTTTTGTTCCAGACACTGGG	-	Foulongne-Oriol et al. 2014	GBEJ01006328	Foulongne-Oriol et al. 2014	scaffold_10	IX	318821	321146	226436
ES53	2	AGAACATTTTCCGTCCGTTC	CACTTGCTATACCGCCTTCA	-	Foulongne-Oriol et al. 2014	GBEJ01003173	Foulongne-Oriol et al. 2014	unassigned	-	-	-	-
ES59	12	GCTGAACAGGAGTAGGAGGC	TTTCTCGTAGAAGCAGGGGT	-	Foulongne-Oriol et al. 2014	GBEJ01004046	Foulongne-Oriol et al. 2014	unassigned	-	-	-	-
ES61	8	GGCTCGCCAGTGCTAATAAC	AACGCCTTCTTCAAACGACT	-	Foulongne-Oriol et al. 2014	GBEJ01004073	Foulongne-Oriol et al. 2014	unassigned	-	-	-	-
**PRS003**	7	CCCAAAGATTTCTCCAACCA	AAATCCCAACTTTGCGTCAC	*Hae* III	this study	GBEJ01009471	Foulongne-Oriol et al. 2014	scaffold_14	IV	493735	495750	195831
**PRS004**	3	AGCCTTGGCGATGTCTATGT	TCGTCTTGGCTTCAAGGACT	*Taq* I	this study	GBEJ01009394	Foulongne-Oriol et al. 2014	scaffold_2	III	1672874	1674342	190666
**PRS009**	4	ATCTCCGATCACATCCTTGC	CAACCTCCGCTTACGAGTTC	*Hpa* II	this study	GBEJ01009914	Foulongne-Oriol et al. 2014	scaffold_7	VII	543213	547207	193606
PRS011	5	TAGCAGGCCATCTGTCATGT	AACTTGACCGTGTTCTTGGTG	*Nde* II	this study	GBEJ01010077	Foulongne-Oriol et al. 2014	scaffold_13	XI	564917	565507	195996
**PRS015**	6	GCTGACCTTCAGTTCGGAGT	CAGAGGCGGGTAGCAAATAG	*Hpa* II	this study	GBEJ01008876	Foulongne-Oriol et al. 2014	scaffold_1	I	2498158	2500175	189950
**PRS016**	12	CCGTCAAGGTCCTCAGTGAT	TTTAGTGCTCATGGCAGCAG	*Nde* II	this study	GBEJ01009029	Foulongne-Oriol et al. 2014	scaffold_9	X	1326408	1327402	194648
PRS018	6	GGCCTTTGGGATAACACTGA	AGAACCGAGAACGACAATCG	*Hha* I	this study	GBEJ01000098	Foulongne-Oriol et al. 2014	unassigned	-	-	-	-
**PRS019**	6	AAATGTGAAAGTCGCCAAGG	CACTTTGAGGCCTTCGTGTT	*Hpy* CH4V	this study	GBEJ01000444	Foulongne-Oriol et al. 2014	scaffold_1	I	1103807	1104645	62276
**PRS022**	3	GATGCCGTCGTAAGGAGAAA	ACAGTGTTGACCACGGTCTG	*Nde* II	this study	GBEJ01002680	Foulongne-Oriol et al. 2014	scaffold_2	III	3044948	3045445	177002
**PRS025**	7	CGTATTTGCGATGCCTCAGT	CGCTTGTATTGACGGTTTGTT	*Hae* III	this study	GBEJ01007792	Foulongne-Oriol et al. 2014	scaffold_4	IV	752135	752829	191764
**PRS029**	13	AGAGGGCTGATGGGAGTTTT	GAGTTTCCTCCCGACGAGTT	*Hinf* I	this study	GBEJ01007599	Foulongne-Oriol et al. 2014	scaffold_12	XII	656041	656776	210981
**PRS034**	8	GGCGTTTGTACCTGTCCACT	CTCTGGTATCGGGATTGGAA	*Taq* I	this study	GBEJ01006282	Foulongne-Oriol et al. 2014	scaffold_3	V	1882739	1884677	191453
**PRS036**	7	CCATAGCCCTGAGTAGCAACA	TGAAGTGATGCCTTCCCAAT	*Rsa* I	this study	GBEJ01006566	Foulongne-Oriol et al. 2014	scaffold_4	IV	1440080	1440782	135514
**PRS038**	9	GCGTGTGTGTGTGTTTGCTA	TTTGAAGTAGCCGCTTTGGT	*Hha* I	this study	GBEJ01003102	Foulongne-Oriol et al. 2014	scaffold_11	XIII	1124511	1125084	210545
**PRS041**	1	CCCCTTCATTATTGCTCGAA	GTTTTGAGTCCGCAGCTTTC	*Hpa* II	this study	GBEJ01004209	Foulongne-Oriol et al. 2014	scaffold_6	VI	676763	677406	143539
**PRS042**	10	ATGGGCGACTTATTCAAACG	AAAACATGTGTGCGAGCAAG	*Alu* I	this study	GBEJ01009421	Foulongne-Oriol et al. 2014	scaffold_13	XI	478713	482067	211439
**PRS045**	13	CATGTCCTACTCGGCGATTT	TTCTTAGCGTCAAGGCTGGT	*Nde* II	this study	GBEJ01009489	Foulongne-Oriol et al. 2014	scaffold_12	XII	541582	543754	195366
**PRS047**	4	ACGCGCCATAATTCAAAATC	GCGTTCTGCTCACATGAAGA	*Nde* II	this study	GBEJ01009166	Foulongne-Oriol et al. 2014	scaffold_7	VII	1650420	1652511	193941
**PRS049**	3	CCGAACGGTTCCATGACTAT	TGTGGTCTCGCTTCATCTTG	*Hinf* I	this study	GBEJ01009154	Foulongne-Oriol et al. 2014	scaffold_2	III	861068	863192	190396
**PRS052**	7	GCAAAAGACTGGGTCACCAT	TCGAGGTAGGATGCGATACC	*Nde* II	this study	GBEJ01008132	Foulongne-Oriol et al. 2014	scaffold_14	IV	271083	273412	195772
**PRS054**	6	GTCTTGAGGTGCGAGGCTAC	CCATGTTCTTGGCGAAAGAT	*Rsa* I	this study	GBEJ01009281	Foulongne-Oriol et al. 2014	scaffold_1	I	1688313	1689656	189667
**PRS055**	9	GCTCGTTTCCAATCTTCGAG	CACATCGCTACCCGTACCTT	*Mse* I	this study	GBEJ01009267	Foulongne-Oriol et al. 2014	scaffold_11	XIII	1230568	1232037	195231
**PRS060**	5	TTCGGTCTCGCTAGGTCAGT	AACGGTAATGCGTTTCTTCG	*Hha* I	this study	GBEJ01009990	Foulongne-Oriol et al. 2014	scaffold_8	VIII	1246877	1248083	194216
**PRS062**	13	GTTGTGCCTGCTCATTACGA	TAAGAACATCGCGATCCACA	*Taq* I	this study	GBEJ01009662	Foulongne-Oriol et al. 2014	scaffold_12	XII	529955	531152	210909
**PRS063**	2	TCGAATGTTGTCAAGCGAGT	CCATTGCTTGGAGTCTGGAG	*Taq* I	this study	GBEJ01008433	Foulongne-Oriol et al. 2014	scaffold_5	II	1871654	1873696	178530
**PRS072**	11	TCTTCCCCAGTAAGCCATTG	TGGTGTCACTGGTCCCATTA	*Nde* II	this study	GBEJ01009317	Foulongne-Oriol et al. 2014	scaffold_10	IX	1329337	1330113	209917

Locus name	LG on *A. subrufescens* map	Forward primer	Reverse Primer	Endonuclease	reference for primer design	designed from	reference for the sequence	Mapping on *A. bisporus* H97 V2.0	Chromosome *A. bisporus*	Mapping-start	Mapping-end	Homolog gene ID in *A. bisporus* genome
**PRS073**	8	AATATGACGCTCGGCAGTTC	AGGGCCTGTCCTGTCTACCT	*Rsa* I	this study	GBEJ01009910	Foulongne-Oriol et al. 2014	scaffold_3	V	1183780	1184866	191242
**PRS075**	8	TCATCTTTTGGGGCGACTAC	GAAGACACCACCCAGGAAAA	*Hae* III	this study	GBEJ01009793	Foulongne-Oriol et al. 2014	scaffold_3	V	832658	833592	218071
**PRS088**	6	CTCGCAATTAGCTTCCAAGG	CGGTTGTCCAAGATCAAGGT	*Hha* I	this study	GBEJ01008187	Foulongne-Oriol et al. 2014	scaffold_1	I	850846	853302	62238
**PRS091**	6	CCGAGAGTTAGAGGGGGAAG	TTGGTTTATCCAGGCTCGTT	*Hae* III	Thongklang et al. 2014	GBEJ01004314	Foulongne-Oriol et al. 2014	scaffold_1	I	903637	904177	213738
**PRS094**	6	GACGGTGCCTAAACACAACA	TGCCATCAACATACCGCTAA	*Hinf* I	this study	GBEJ01006100	Foulongne-Oriol et al. 2014	scaffold_1	I	998926	1000072	189424
**PRS095**	6	CGCAACTTGAATAACGCTCA	TATGCGCGAGATTACGACTG	*Hinf* I	this study	GBEJ01007393	Foulongne-Oriol et al. 2014	scaffold_1	I	12142	14030	189140
**PRS101**	6	CGGAGGGTGAAATTCAGAAA	TAGACATGCCACGATTGGAA	*Hae* III	this study	GBEJ01007029	Foulongne-Oriol et al. 2014	scaffold_1	I	478079	479495	189260
**PRS102**	6	TTAATGGCTCCGATGAAAGG	GCAGCAAAACTCCAACCTTC	*Rsa* I	this study	GBEJ01008988	Foulongne-Oriol et al. 2014	scaffold_1	I	126162	126950	175592
**PRS108**	3	GATCCCGAACGTTCTTTGAA	CGCTGTTGACAGCAAGAATG	*Hae* III	this study	GBEJ01000019	Foulongne-Oriol et al. 2014	scaffold_2	III	2292444	2293011	217231
**PRS109**	7	TCGTGTTTCTTTGTTGCAATCT	CCGCAAGCTGTTGAGAATTT	*Hpa* II	this study	GBEJ01000008	Foulongne-Oriol et al. 2014	scaffold_4	IV	597387	598000	184435
**PRS113**	6	TAGTTTAGGGCGCATCAACC	CCTCCAACCAACACTCATCC	*Hsp* 92 II	this study	GBEJ01000012	Foulongne-Oriol et al. 2014	scaffold_1	I	373143	373641	147275
**PRS114**	3	CGGTGAGCGTGAAAATATGA	AGGCACCCAATTCACAAAAG	*Hha* I	this study	GBEJ01000027	Foulongne-Oriol et al. 2014	scaffold_2	III	1208585	1209134	64815
**PRS121**	3	GGAAGAGTCTCGCTCCAGAA	AGGAATGACCTGACTGTAGCC	*Hpa II*	this study	GBEJ01000072	Foulongne-Oriol et al. 2014	scaffold_2	III	1121753	1122435	183212
**PRS123**	4	AGTTGTTTCCAGCGTCTTCC	ACCAACGCCAGCAGCTAATA	*Hha* I	this study	GBEJ01000093	Foulongne-Oriol et al. 2014	scaffold_7	VII	742418	743030	193658
**PRS125**	3	GTTAGAGCCCATGGCAAGAA	GGACAACACAGTCGTGATTAAC	*Mse* I	this study	GBEJ01000104	Foulongne-Oriol et al. 2014	scaffold_2	III	926844	927412	216197
**PRS129**	2	ATAAAGGACCAAGCCCAACC	CCTTCTTAGCCTGCTTCGTG	*Nde* II	this study	GBEJ01000133	Foulongne-Oriol et al. 2014	scaffold_5	II	1320771	1321273	118043
**PRS132**	10	TATACGAGCCAGTTGCGAGA	ATATTGATGGGACGGTGGAA	*Alu* I	this study	GBEJ01000195	Foulongne-Oriol et al. 2014	scaffold_13	XI	361863	362531	211380
**PRS139**	12	CGGACGATCCAAAGAAGAAG	GAAGTGTGCCTCGAGGTTGT	*Alu* I	this study	GBEJ01000388	Foulongne-Oriol et al. 2014	scaffold_9	X	1024120	1024812	194589
**PRS141**	15	CCACAAGTACCTTCGTTCACAA	CCAGCAGGGTTTGGTAAGAG	*Alu* I	this study	GBEJ01000401	Foulongne-Oriol et al. 2014	scaffold_10	IX	545559	546122	194858
**PRS144**	11	CAAGTTCGCCTTCGAATGTT	GGCTCGACGAACACCCTAT	*Hae* III	this study	GBEJ01000648	Foulongne-Oriol et al. 2014	scaffold_10	IX	859682	860201	209738
**PRS145**	12	CAAATTTCATCAGCCCTCCT	CCCCAACTCGTGTGTCAGT	*Hinf* I	this study	GBEJ01000670	Foulongne-Oriol et al. 2014	scaffold_9	X	672081	672597	120641
**PRS149**	9	AAATATCCCCATGCGCACTT	CGACTGCATCAACCGCTTAC	*Hae* III	this study	GBEJ01000838	Foulongne-Oriol et al. 2014	scaffold_11	XIII	669430	670121	76781
**PRS159**	2	AAGTGACAAGATCCCCGACA	AGGTTAGTAGCTGGCGTTGG	*Nde* II	this study	GBEJ01000495	Foulongne-Oriol et al. 2014	scaffold_16	II	412280	412834	229642
**PRS160**	4	CACTGAACGTGACCTGGAGA	AGGGTTTTCGGATGACATTG	*Hae III*	this study	GBEJ01000570	Foulongne-Oriol et al. 2014	scaffold_7	VII	232331	232917	193525
**PRS162**	4	ATGGTGCTGTTGCCGTTATT	ATCAACATCGGGGTTCAATC	*Rsa I*	this study	GBEJ01000708	Foulongne-Oriol et al. 2014	scaffold_7	VII	417745	418407	119279
**PRS165**	4	GAGCTCTGCAGGTCCATCTC	AGTCGACAAGTGCTCCAAGG	*Rsa* I	this study	GBEJ01001466	Foulongne-Oriol et al. 2014	scaffold_7	VII	1441668	1442267	186649
**PRS166**	4	TCCCTACGTCGGCTCATTAC	AATCCATGCCTGTCGTTCTT	*Hinf* I	this study	GBEJ01001657	Foulongne-Oriol et al. 2014	scaffold_7	VII	1471447	1471980	224431
PRS168	6	TCGGGTTTGTTTGTGACATC	TACAAGAAGGCGGAACTTCG	*Hae* III	this study	GBEJ01000004	Foulongne-Oriol et al. 2014	unassigned	-	-	-	-
PRS171	14	GCGCCCTCAGTATATTTGGA	TACCTTCGGCTCGTGAGAGT	*Rsa* I	this study	GBEJ01000018	Foulongne-Oriol et al. 2014	unassigned	-	-	-	-
PRS175	16	TAATTGCGGCATCCAATGTA	TCTCTCACCCTTGACGAAGC	*Hpa* II	this study	GBEJ01000034	Foulongne-Oriol et al. 2014	unassigned	-	-	-	-
**PRS176**	7	TGCTGTTCCGAGAAACTGTG	TGCCCAGTTCATTAACAACG	*Hpa* II	this study	GBEJ01000035	Foulongne-Oriol et al. 2014	scaffold_4	IV	1355019	1355621	220085
PRS177	10	CGAGGCTCGAACTTAGATGG	AGCCAACGATAATGGAATCG	*Mse* I	this study	GBEJ01000039	Foulongne-Oriol et al. 2014	unassigned	-	-	-	-
PRS178	1	AGGGCTTTGCTGATTCTCAA	CGGGCATCGAGTACATTCTT	*Hsp* 92 II	this study	GBEJ01000044	Foulongne-Oriol et al. 2014	unassigned	-	-	-	-
**PRS181**	11	GCCGGTATGCCTGGAAGTA	CTCAATGGCAAAACTCACGA	*Hha* I	this study	GBEJ01008785	Foulongne-Oriol et al. 2014	scaffold_10	IX	875674	876552	153347
**PRS188**	5	GCACGATAGGGAACGTGTGA	TGCAGTAGCAGCAACAAAGG	*Hha* I	this study	GBEJ01009811	Foulongne-Oriol et al. 2014	scaffold_8	VIII	689577	690314	189423
**PRS190**	11	ATGGGCCATCGGAATATACA	ACCATTCCCACTTTGACAGC	*Hinf* I	this study	GBEJ01007939	Foulongne-Oriol et al. 2014	scaffold_10	IX	748566	750700	138510
**PRS191**	5	GAGTTCGCCAGCGAGAATAC	CTAAGCCAAAGTGGCGAGAC	*Rsa* I	this study	GBEJ01010002	Foulongne-Oriol et al. 2014	scaffold_8	VIII	741716	743644	194113
**PRS202**	5	ATCGCATCTTGTCGATTTCC	GGCACCTTCCAGCAATATGT	*Rsa* I	this study	GBEJ01009246	Foulongne-Oriol et al. 2014	scaffold_8	VIII	1152783	1154205	194191
**PRS204**	5	GTTTCCCATCGACACGATCT	GATCCTGACGCGACTTTCTC	*Hinf* I	this study	GBEJ01009032	Foulongne-Oriol et al. 2014	scaffold_8	VIII	1186735	1187448	186955
**PRS206**	2	CAACAAATTCCGTCACATCG	GCGACTTTGATTTCGGGTAA	*Hae* III	this study	GBEJ01009428	Foulongne-Oriol et al. 2014	scaffold_5	II	2231478	2232779	205248
**PRS213**	1	GACCTGACAACGGGTTATGC	ATTCCTCACCTCGGACACAC	*Hpa* II	this study	GBEJ01009652	Foulongne-Oriol et al. 2014	scaffold_6	VI	1664474	1665178	193382
PRS215	2	CTACTGCGCACACAACGATT	ACTCCCTCCTCTCCTTCACC	*Rsa* I	this study	GBEJ01008574	Foulongne-Oriol et al. 2014	unassigned	-	-	-	-
PRS217	10	CTCAACCTGCTCACGAACAA	GCATGTCATGTCGTCGAGTT	*Hinf* I	this study	GBEJ01009271	Foulongne-Oriol et al. 2014	unassigned	-	-	-	-
PRS219	10	ACCCGTGACCAGAGGTACTG	TTGATGACCTCCCCGATTAC	*Taq* I	this study	GBEJ01008346	Foulongne-Oriol et al. 2014	unassigned	-	-	-	-

Locus name	LG on *A. subrufescens* map	Forward primer	Reverse Primer	Endonuclease	reference for primer design	designed from	reference for the sequence	Mapping on *A. bisporus* H97 V2.0	Chromosome *A. bisporus*	Mapping-start	Mapping-end	Homolog gene ID in *A. bisporus* genome
**PRS227**	2	TCTACCAGCCGTTTGGAAAG	CACCACCAACCAAGGAAACT	*Hsp* 92 II	this study	GBEJ01009100	Foulongne-Oriol et al. 2014	scaffold_5	II	1804790	1805492	192824
**PRS228**	2	CCCTTGACGGGAATCATAAA	ACCCGGTGATAGTGACGAAG	*Rsa* I	this study	GBEJ01009040	Foulongne-Oriol et al. 2014	scaffold_5	II	288530	289574	203925
PRS233	1	GAGCCCCAGAATGTTCGATA	CACCAGGCAGTGATCAGAGA	*Hpa* II	this study	GBEJ01008341	Foulongne-Oriol et al. 2014	unassigned	-	-	-	-
**PRS234**	11	TAGGCAATCAGGCAAAGGTT	TGCGAGAACATGAACGAGAC	*Hinf* I	this study	GBEJ01008836	Foulongne-Oriol et al. 2014	scaffold_10	IX	97711	98627	153136
**PRS235**	16	TGGCGAAAATGATGATGAAA	GAGCGGGAAGATGGATGATA	*Hinf* I	this study	GBEJ01010033	Foulongne-Oriol et al. 2014	scaffold_8	VIII	1725263	1726430	187111
**PRS239**	2	TACAGTACGCTGCCAAATCG	TGAACAAGCACCTGAAAGCA	*Alu* I	this study	GBEJ01009420	Foulongne-Oriol et al. 2014	scaffold_5	II	215643	216886	178164
**PRS240**	1	ATTGCAGATCTTCCCGCTAA	TCGTGTGAAGGCATTGTACC	*Hinf* I	this study	GBEJ01008462	Foulongne-Oriol et al. 2014	scaffold_6	VI	1988077	1989115	193452
**PRS241**	1	AGCGCGAAGATTTTTCTGAC	AAACCGCAACTTCACTGGTC	*Hsp* 92 II	this study	GBEJ01008219	Foulongne-Oriol et al. 2014	scaffold_6	VI	1325976	1326555	223047
**PRS246**	2	CACTACCCACCGACGAAGAT	CGTCAAACCATTCATGTCCA	*Nde* II	this study	GBEJ01006977	Foulongne-Oriol et al. 2014	scaffold_5	II	795968	796623	178316
**PRS250**	1	TTATTGTCGATCAAGTGGGA	TCCGAATATGAATGAGAAACCA	*Nde* II	this study	GBEJ01006795	Foulongne-Oriol et al. 2014	scaffold_6	VI	690992	691526	178776
**PRS254**	1	CCGTCTTCTGGCGAGTACAG	CAGATGCAGGATCACGTCTG	*Hpa* II	this study	GBEJ01007854	Foulongne-Oriol et al. 2014	scaffold_6	VI	1353126	1353749	223067
**PRS256**	5	ACACTCGCACCCTTCTTCCT	TAATCCTCGGGTGTCTGGTT	*Nde* II	this study	GBEJ01007114	Foulongne-Oriol et al. 2014	scaffold_8	VIII	1101380	1102917	120153
PRS267	3	GGTCGCTCCCTCATTAAACA	TCGGTAAACTCTGGTTCGTG	*Alu* I	this study	GBEJ01006177	Foulongne-Oriol et al. 2014	scaffold_15	XI	100044	101049	212249
PRS269	10	TTTCGCATTACACGCTTCAC	AAGCCAGCAAACTTCGTCAT	*Rsa* I	this study	GBEJ01006377	Foulongne-Oriol et al. 2014	unassigned	-	-	-	-
**PRS279**	11	TTTCGCATTACACGCTTCAC	AAGCCAGCAAACTTCGTCAT	*Rsa* I	this study	GBEJ01009514	Foulongne-Oriol et al. 2014	scaffold_10	IX	1512827	1514061	195012
**Rpb2**	6	GTCTTCCGGAGTCATCGAAA	GCCAAACCTCGTCAACTCCA	*Hpa* II	this study	KJ541801	Thongklang et al. 2014	scaffold_1	I	871331	871889	113824
SubSSR10	3	AGCACAACGGGGATCTAATG	CAGGAAGGAACTCTTATTAGGGAA	-	Foulongne-Oriol et al. 2012	JQ901361	Foulongne-Oriol et al. 2012	unassigned	-	-	-	-
SubSSR14	4	TCGGGGTAGGATACAGATGC	GAAACCCTTTTATTCGTCACTG	-	Foulongne-Oriol et al. 2012	JQ901239	Foulongne-Oriol et al. 2012	unassigned	-	-	-	-
**SubSSR21**	16	CTCAAACCAAACCTCCCAAA	AAGCCGATGCGATATTTACG	-	Foulongne-Oriol et al. 2012	JQ901126	Foulongne-Oriol et al. 2012	scaffold_8	VIII	1658002	1658173	120329
SubSSR22	11	ACACCTCCAAGATGTCGTCC	GATGGAGAGGAGGGATAGGG	-	Foulongne-Oriol et al. 2012	JQ901144	Foulongne-Oriol et al. 2012	unassigned	-	-	-	-
SubSSR27	14	TCACACCCAATCAGCGAATA	TACGTCAACGCGAATTGTTC	-	Foulongne-Oriol et al. 2012	JQ901143	Foulongne-Oriol et al. 2012	unassigned	-	-	-	-
SubSSR36	5	GGGATCCGGAGATAGGACTT	GGGATGACGGTTTGGTTTTT	-	Foulongne-Oriol et al. 2012	JQ901145	Foulongne-Oriol et al. 2012	unassigned	-	-	-	-
SubSSR47	4	GTCGTACGCCGTCTCCTATC	GCCGTCGAAGTGAATACCAT	-	Foulongne-Oriol et al. 2012	JQ901326	Foulongne-Oriol et al. 2012	unassigned	-	-	-	-
**SubSSR50**	1	GACAAGAAGCGAAGGAGTGC	ATGGCAGTAATCGGGAACAG	-	Foulongne-Oriol et al. 2012	JQ901140	Foulongne-Oriol et al. 2012	scaffold_6	VI	462928	462993	205497
**SubSSR52**	10	CTTCGCCCAGCTTTGTAGTC	ACAGAATCACCGCAATCCTC	-	Foulongne-Oriol et al. 2012	JQ901191	Foulongne-Oriol et al. 2012	scaffold_13	XI	780945	781087	188477
**SubSSR54**	7	ACCGTCATCCAGTCGTTAGG	CCATGGTCGGCTTCTACACT	-	Foulongne-Oriol et al. 2012	JQ901316	Foulongne-Oriol et al. 2012	scaffold_4	IV	677027	677089	116920
**SubSSR56**	3	AAGTGACTCGCTCAATGCCT	TACCCATAGGTGAAAGGGCA	-	Foulongne-Oriol et al. 2012	JQ901170	Foulongne-Oriol et al. 2012	scaffold_2	III	1311146	1311082	199581
**SubSSR59**	2	TGGCTGCATCTTGATCTGAC	TGTAGCAGTGGAAGGTGGTG	-	Foulongne-Oriol et al. 2012	JQ901372	Foulongne-Oriol et al. 2012	scaffold_5	II	1904932	1904729	192859
SubSSR65	9	AGCGGCGCAACTGTATAGC	GAATGCCAGAAGAGTGAGGC	-	Foulongne-Oriol et al. 2012	cons86 in SRA050786	Foulongne-Oriol et al. 2012	unassigned	-	-	-	-
**SubSSR66**	1	CGCCATACTCTGCTTCACCT	GGCGAAGTCTGAGGAGTCAA	-	Foulongne-Oriol et al. 2012	JQ901204	Foulongne-Oriol et al. 2012	scaffold_6	VI	1356833	1356893	151584
SubSSR68	1	AACATCCTTTCCAAACACGC	GTTGATTGATTTCCAACGCA	-	Foulongne-Oriol et al. 2012	JQ901370	Foulongne-Oriol et al. 2012	unassigned	-	-	-	-
**SubSSR76**	8	GATACGGCGATGGAACTTGT	CAACTCCTCAAGCTCAGTTCAA	-	Foulongne-Oriol et al. 2012	JQ901179	Foulongne-Oriol et al. 2012	scaffold_3	V	1555293	1555407	201553
SubSSR80	3	CCGCACTCATTTAATTTTGC	GAGGGCTGAGCATAAAGCAC	-	Foulongne-Oriol et al. 2012	JQ901208	Foulongne-Oriol et al. 2012	unassigned	-	-	-	-
SubSSR83	12	CATCGAATCGGAAGACGAAT	CTCAACTCCCACACCCACTT	-	Foulongne-Oriol et al. 2012	JQ901374	Foulongne-Oriol et al. 2012	unassigned	-	-	-	-
**SubSSR86**	8	GTGTCTCTATAGCGGCCTCG	AGGACAGCTGTGTCTGCTCA	-	Foulongne-Oriol et al. 2012	JQ901182	Foulongne-Oriol et al. 2012	scaffold_3	V	2493884	2493767	177544
**SubSSR91**	7	ATGGACACATACCGCAGGTC	TGGCCAGATATTTTGCATCA	-	Foulongne-Oriol et al. 2012	JQ901221	Foulongne-Oriol et al. 2012	scaffold_4	IV	63954	63798	191599
**SubSSR93**	13	AACGTCGTCGACAACAACC	GCTGAAGATAGAGGCGATGG	-	Foulongne-Oriol et al. 2012	JQ901115	Foulongne-Oriol et al. 2012	scaffold_12	XII	661316	661398	180899

Table B : The 7 AFLP primers combinations used for the genotyping of *A. subrufescens* progeny

	Eco RI primer +0<br>GACTGCGTACCAATTC	*Mse* I primer +0<br>GATGAGTCCTGAGTAA	marker code
+2 Primer extension	GC	AG	GCAG
	GC	AA	GCAA
	CA	AC	CAAC
	AG	TA	AGTA
	AG	GC	AGGC
	AC	TA	ACTA
	AC	CG	ACCG

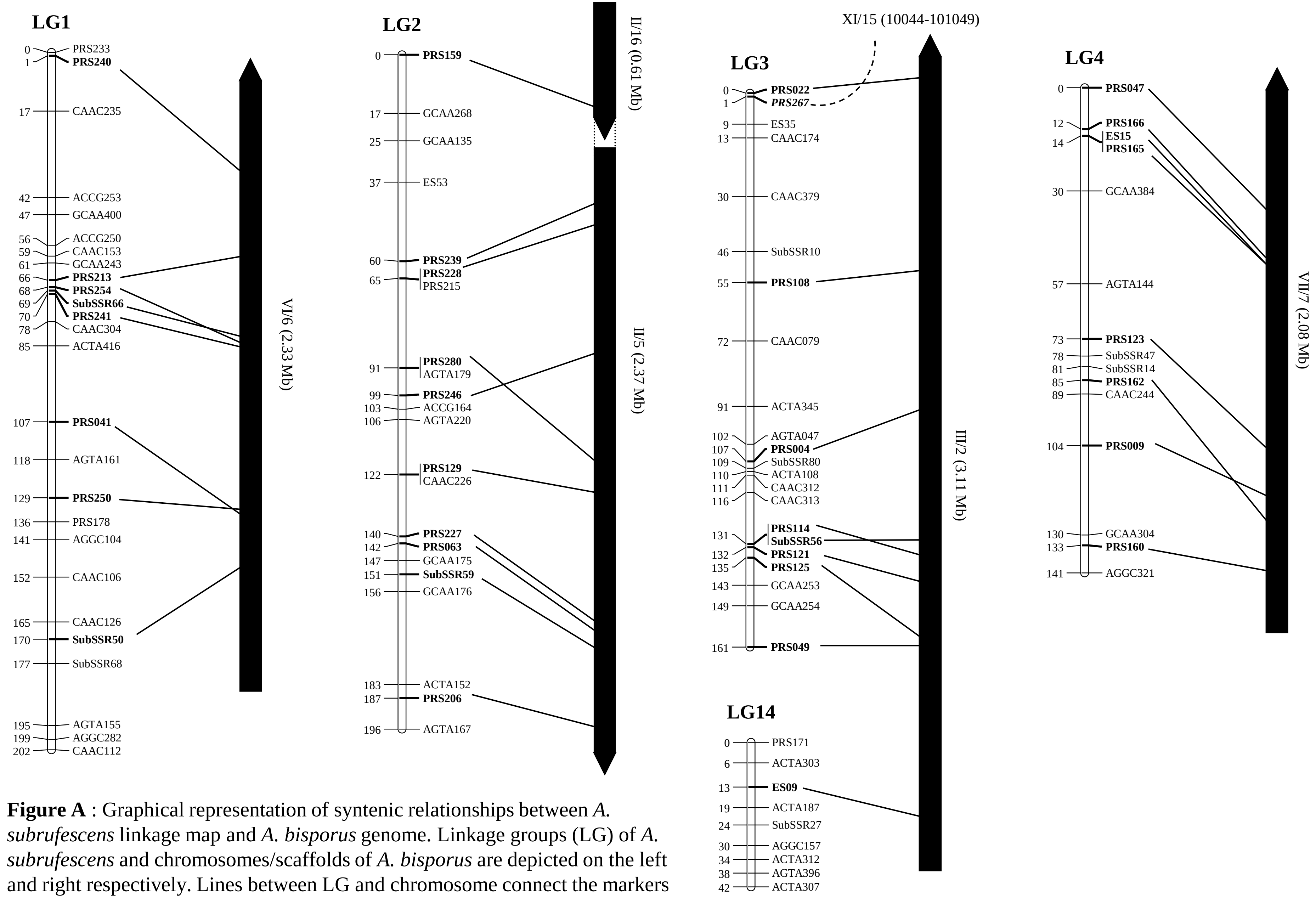

Figure A : Graphical representation of syntenic relationships between *A. subrufescens* linkage map and *A. bisporus* genome. Linkage groups (LG) of *A. subrufescens* and chromosomes/scaffolds of *A. bisporus* are depicted on the left and right respectively. Lines between LG and chromosome connect the markers mapped on *A. subrufescens* to the position of their homologs on the *A. bisporus* genome. This figure is a linear version of the Circos Fig.2 presented in the manuscript.

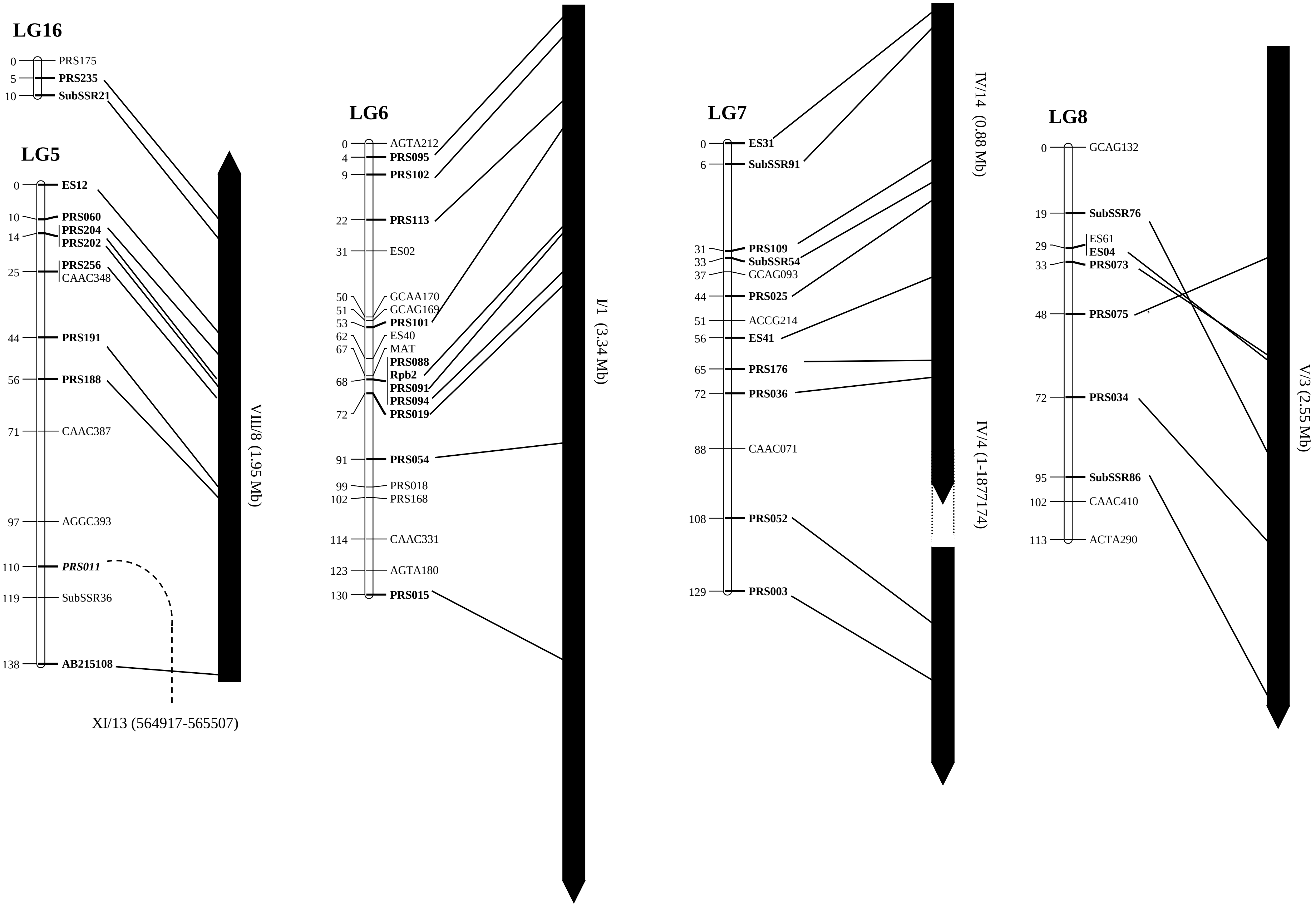

LG16
0 PRS175
5 PRS235
10 SubSSR21
LG5
0 ES12
10 PRS060
14 PRS204
PRS202
25 PRS256
CAAC348
44 PRS191
56 PRS188
71 CAAC387
97 AGGC393
110 PRS011
119 SubSSR36
138 AB215108
VIII/8 (1.95 Mb)
XI/13 (564917-565507)
LG6
0 AGTA212
4 PRS095
9 PRS102
22 PRS113
31 ES02
50 GCAA170
51 GCAG169
53 PRS101
62 ES40
67 MAT
PRS088
68 Rpb2
PRS091
PRS094
72 PRS019
91 PRS054
99 PRS018
102 PRS168
114 CAAC331
123 AGTA180
130 PRS015
I/1 (3.34 Mb)
LG7
0 ES31
6 SubSSR91
31 PRS109
33 SubSSR54
37 GCAG093
44 PRS025
51 ACCG214
56 ES41
65 PRS176
72 PRS036
88 CAAC071
108 PRS052
129 PRS003
IV/14 (0.88 Mb)
IV/4 (1-187174)
LG8
0 GCAG132
19 SubSSR76
29 ES61
ES04
33 PRS073
48 PRS075
72 PRS034
95 SubSSR86
102 CAAC410
113 ACTA290
V/3 (2.55 Mb)

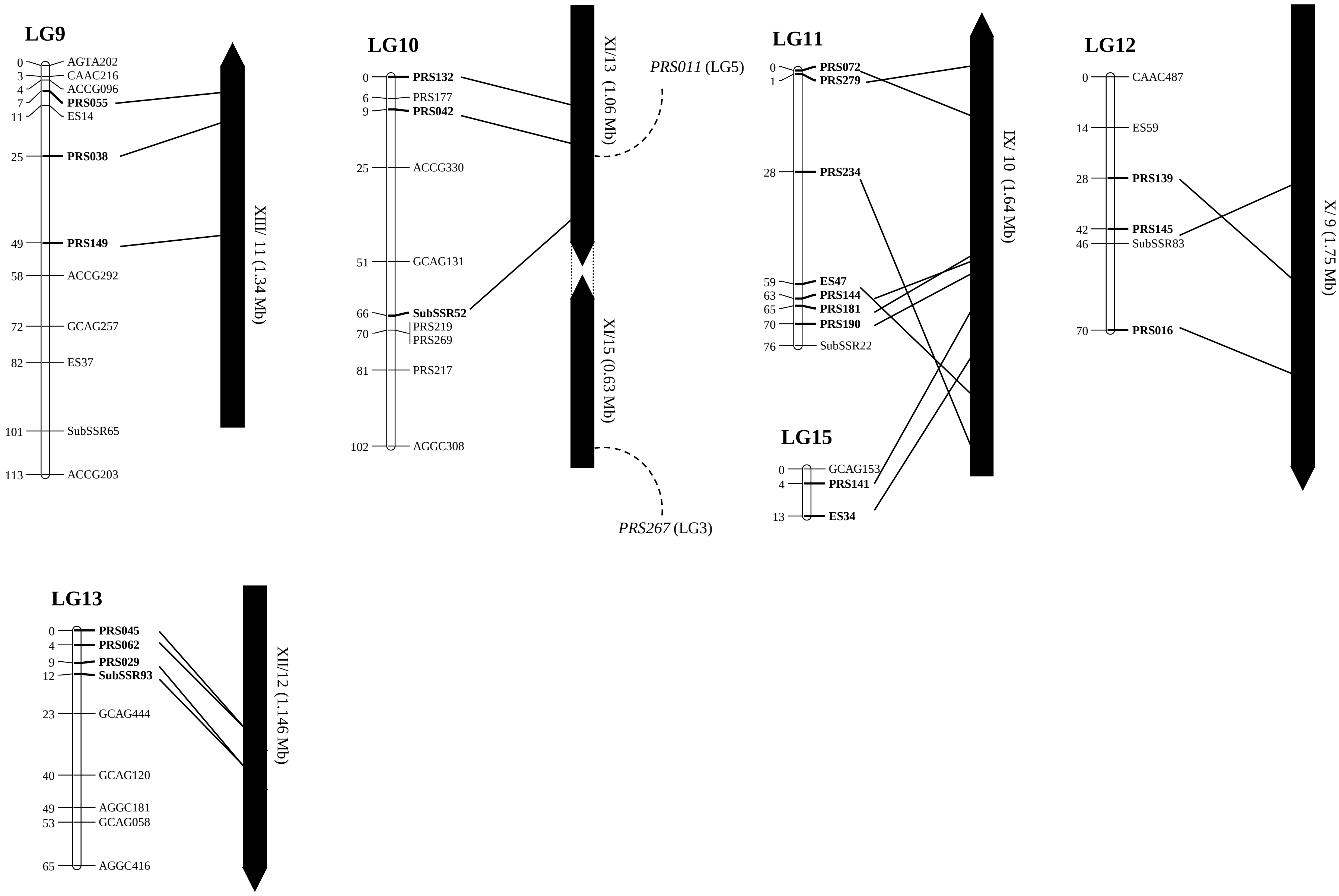

LG9
0 AGTA202
3 CAAC216
4 ACCG096
7 PRS055
11 ES14
25 PRS038
49 PRS149
58 ACCG292
72 GCAG257
82 ES37
101 SubSSR65
113 ACCG203
XIII/ 11 (1.34 Mb)
LG10
0 PRS132
6 PRS177
9 PRS042
25 ACCG330
51 GCAG131
66 SubSSR52
70 PRS219
PRS269
81 PRS217
102 AGGC308
XI/13 (1.06 Mb)
XI/15 (0.63 Mb)
PRS011 (LG5)
PRS267 (LG3)
LG11
0 PRS072
1 PRS279
28 PRS234
59 ES47
63 PRS144
65 PRS181
70 PRS190
76 SubSSR22
IX/ 10 (1.64 Mb)
LG15
0 GCAG153
4 PRS141
13 ES34
LG12
0 CAAC487
14 ES59
28 PRS139
42 PRS145
46 SubSSR83
70 PRS016
X/ 9 (1.75 Mb)
LG13
0 PRS045
4 PRS062
9 PRS029
12 SubSSR93
23 GCAG444
40 GCAG120
49 AGGC181
53 GCAG058
65 AGGC416
XII/12 (1.146 Mb)
